# Spleen-Kidney Supplementing Formula Alleviates Renal Fibrosis in Diabetic Rats via TGF-*β*1-miR-21-PTEN Signaling Pathway

**DOI:** 10.1155/2018/3824357

**Published:** 2018-12-06

**Authors:** Chunyu Tian, Ya Wang, Hong Chang, Ji'an Li, Xiaojin La

**Affiliations:** Traditional Chinese Medical College, North China University of Science and Technology, Tangshan 063210, China

## Abstract

**Background:**

Spleen-Kidney Supplementing Formula (SKSF), composed of 6 raw Chinese herbs and proposed based on the therapeutic principle of supplementing spleen-kidney and clearing the extra heat of stomach-lung, is effective in the treatment of type 2 diabetes mellitus (T2DM).

**Aim:**

This study aims to investigate the mechanism of SKSF to alleviate renal fibrosis in diabetic rats.

**Methods:**

T2DM model was induced by high-fat diet and multiple injections of low-dose streptozotocin. After 8-week intervention, samples were collected for detection.

**Results:**

SKSF decreased fasting blood glucose, glycosylated hemoglobin A1c, blood urea nitrogen, uric acid, urea, 24-hour urine protein, and KW/BW ratio, while it increased creatinine clearance rate of T2DM rats. Meanwhile, SKSF attenuated the renal fibrosis and improved the morphology and structure of renal tissue. Furthermore, SKSF significantly reduced the expression level of plasma miR-21 and TGF-*β*1 protein level and increased PTEN protein level.

**Conclusion:**

SKSF could attenuate the renal damage and renal fibrosis induced in T2DM, which may be related to its regulation on the expressions of TGF-*β*1, PTEN, and miR-21.

## 1. Introduction

Type 2 diabetes mellitus (T2DM) is an increasing public health issue and its prevalence has been growing at a high rate [1, 2]. About 425 million patients worldwide are estimated to suffer from T2DM, and some 629 million individuals are projected to develop T2DM by 2045 [1]. T2DM and its complications have high risk of disabilities and mortality [3], and therefore, medical scholars in various countries devote themselves to exploring safe and effective drugs for the prevention and treatment of T2DM.

Diabetic kidney disease (DKD), also named diabetic nephropathy (DN), is one of the most common complications of T2DM, and approximately 20-40% individuals with T2DM will develop. The pathogenesis and natural history of DN is characterized initially by microalbuminuria followed by a progressive decline in glomerular function. The terminal stage of DN is characterized by the development of chronic renal failure which is considered as the main cause of death in diabetics [4]. Therefore, timely and effective treatment for DN is of great significance. Treatment for T2DM patients with DN is challenging, because the decline in renal function impairs the clearance and metabolism of antidiabetic agents and insulin, requiring frequent reassessment of prescriptions. General approach for DN includes glycemic control, hypertension reduction, lipid control, and renal fibrosis inhibition [5].

Transforming growth factor-beta (TGF-*β*) is a multifunctional regulatory polypeptide that controls many aspects of cellular function, including cellular proliferation, differentiation, and apoptosis [6]. TGF-*β* is recognized as the key initiator in the fibrotic process. Experimental studies have shown that high glucose concentration in cell culture medium overactivates TGF-*β*/Smad pathway [7]. The synthesis of type IV collagen (COL-IV), fibronectin (FN), and laminin (LN) increased in renal mesenchymal cells, renal tubular epithelium cells, endothelial cells, and other mesenchymal cells [8], which accelerates the accumulation of extra-cellular matrix (ECM) and forms renal interstitial fibrosis, resulting in renal fibrosis. In vitro experiments confirmed that overexpressed TGF-*β* in renal tubular epithelial cell line mediates Smad3 signaling pathway and increases expression of miR-21 [9]; the latter further stimulates the PI3K/Akt pathway through the regulation of PTEN protein expression, resulting in the activation of downstream kinases which induce the hypertrophy of renal interstitial cells, abnormal deposition of ECM, and apoptosis of podocytes [9].

Traditional Chinese medicine (TCM) has made significant contribution to the prevention and treatment of T2DM in recent years [10], with therapeutic effects on relieving insulin resistance (IR), ameliorating glycometabolism, controlling the occurrence of complications, and improving their prognosis. Through pattern identification, we consider spleen-kidney deficiency as the basic pathogenesis mechanism of diabetes, and dryness-heat, especially lung dryness and stomach fire excess, as its secondary pathogenesis mechanism. Over-intake of fatty and sweet food is the main reason for diabetes. Spleen-Kidney Supplementing Formula (SKSF) was created based on the above pathogenesis mechanisms and the therapeutic principle of supplementing spleen-kidney and clearing the extra heat of stomach-lung. In SKSF, Radix Astragali fortifies the spleen-qi and Fructus Corni tonifies the kidney. Rhizoma Coptidis clears stomach fire and Cortex Mori drains lung heat. Radix Puerariae Lobatae clears heat and promotes fluid production. Herba Eupatorii eliminates dampness. Our previous pharmacological experiments showed that berberine [11, 12] and puerarin [13] are the main effective components in SKSF, for their hypoglycemic effect. In this study, the antidiabetic effect of SKSF was evaluated. In addition, we also observed the influence of SKSF on renal function, plasma miR-21 expression, kidney pathological morphology, and protein expression of TGF-*β*1 and PTEN in T2DM model rats to analyze its mechanism and provided experimental evidence for the development and utilization of SKSF.

## 2. Materials and Methods

### 2.1. Animals

Specific pathogen-free (SPF) Wistar male rats (190±10 g) were purchased from the Animal Center of Academy of Military Medical Sciences (SCXK (Army) 2014-0001, Beijing, China). The rats were maintained in a temperature (20–25°C) and humidity (45%) controlled environment with a 12-hour-light/12-hour-dark cycle in Laboratory Animal Center of North China University of Science and Technology (MY10DXK07; Tangshan, China).

### 2.2. Drugs and Reagents

Radix Astragali, Fructus Corni, Rhizoma Coptidis, Radix Puerariae Lobatae, Cortex Mori, and Herba Eupatorii are ingredients of SKSF, which were purchased from Tongrentang Pharmacy Branch (Tangshan, China). Metformin hydrochloride tablets (500 mg each) were manufactured by Sino-American Shanghai Squib Pharmaceutical Ltd. (Shanghai, China). The kits of glycosylated hemoglobin A1c (HbA1c) assay, creatinine assay, urea assay, blood urea nitrogen (BUN) assay, and uric acid (UA) assay were from Nanjing Jiancheng Bioengineering Institute (Nanjing, China). Primary antibodies of TGF-*β*1 and PTEN were from Beyotime Institute of Biotechnology (Shanghai, China). PV-9001 reagents kit was from Beijing Zhongshan Jinqiao Biotechnology Co., Ltd. (Beijing, China), and Ambion® mirVana^TM^ PARIS kit was from Invitrogen (Carlsbad, USA). The following products were from Applied Biosystems Inc. (Foster, CA, USA): TaqMan® MicroRNA Reverse Transcription kit, TaqMan® MicroRNA assays, and TaqMan® Universal PCR Master Mix.

### 2.3. Main Instruments

Glucose meter (Sinocare Inc., Changsha, China); CHEMRAY 240 automated chemistry analyzer (Raito Inc., Hayward, CA, USA); M200PRO microplate reader (Tecan Group Ltd., Männedorf, Switzerland); Multifuge X1R refrigerated centrifuge (Jouan, France); S1000 thermal cycler (Bio-Rad, USA); and 7500 real-time PCR system (ABI Inc., USA) were used.

### 2.4. T2DM Model Establishment, Grouping, and Drug Administration

Wistar rats were fed for one week to adapt to the environment. Ten rats were randomly selected as the normal group (group N) and fed with standard diet. The remaining rats were fed with high-fat diet (kcal%, containing 60% fat, 20% protein, and 20% carbohydrates from Beijing Huafukang Bioscience Co., Ltd. (Beijing, China)) for 4 weeks. Then the rats were intraperitoneally injected with streptozotocin (STZ) solution (25 mg/kg, twice at weekly intervals) [14]. The rats in group N were injected with the same dose of buffer solution. All rats were fasted for 12 hours before STZ injection. On day 7 after the final injection of STZ, all rats were tested for the levels of fasting blood glucose (FBG) and random blood glucose (RBG) (8:00 A.M.). Fifty-nine rats were considered to be diabetic with FBG *⩾* 11.1 mmol/L or RBG *⩾* 16.7 mmol/L [15]. After exclusion of 9 rats with FBG > 30.0 mmol/L, the remaining 50 diabetic rats were randomly divided into the model group (group M), the metformin group (Group Met), and the SKSF groups of low, medium, and high dose (Group SKSF-l, SKSF-m, and SKSF-h, respectively), with 10 rats in each group. All rats were provided with the corresponding drugs via intragastric administration. According to the drug dosage conversion formula between rats and human, the rat dosage is 6.3 times of human's. Rats in group N and group M were given purified water; rats in Group Met were administrated with metformin hydrochloride suspension 85 mg/kg/d; rats in Groups SKSF-l, SKSF-m, and SKSF-h were provided with alcohol-extracted SKSF (2000, 1000, and 500 mg/kg, respectively). Rats were weighed once a week for adjusting drug dosage during the 8-week intervention period.

### 2.5. Sampling

In the last week of intervention, the urine from all rats was collected for measuring the 24-hour urine protein excretion. After rats were anesthetized, the blood from abdominal aorta was collected and kept in EDTA anticoagulant tube. Kidneys were removed quickly and the left kidney mass was measured after removal of renal capsule. The left kidney was fixed in 10% formaldehyde solution.

### 2.6. Preparation of SKSF Extraction

The ingredients of SKSF extraction were mixed thoroughly (in the ratio of 5:5:4:4:3:2). The optimal extraction technology was as follows: six times the amount of 30% ethanol, heat reflux extraction twice (1.5 hours for each time). The extraction was dehydrated in vacuo (70°C) and ground into powder. The prepared powder was kept at 0-4°C and dissolved with purified water into suspension of different concentrations.

### 2.7. Biochemical Analysis

Blood samples were centrifuged (2000 rpm, 15 minutes, 4°C). Serum concentration of BUN, urea, creatinine, and UA were determined with kits according to manufactures' instructions. HA1c was detected by the microplate reader, and fasting blood glucose (FBG) was detected by the glucose meter.

### 2.8. H&E Staining and Masson's Trichrome Staining on Renal Tissues

Renal tissues were fixed in 10% formaldehyde solution. After that, the tissues were dehydrated, paraffin-embedded, and sectioned into 4 *μ*m thick slices. H&E staining was performed to visualize the histological structure, and Masson's trichrome staining was used to observe the fibrosis.

### 2.9. Expressions of TGF-*β*1 and PTEN via Immunohistochemistry

4 *μ*m thick sections were obtained from paraffin-embedded tissue blocks and were placed on a silanized glass slide. The sections were deparaffinized and shifted for antigen retrieval. Afterwards, the samples were immersed in 3% hydrogen peroxide to block endogenous peroxidase activity. After treatment with normal serum, the sections were incubated with the anti-TGF-*β*1 (1:200) and anti-PTEN (1:200) primary antibodies in a moist chamber at room temperature. Sections were then treated with the Two-Step IHC Detection Reagent (PV-9001) and visualized by incubating with DAB. All sections were analyzed using Image-Pro Plus 6.0 at ×400 magnification to quantify protein expression, and 10 random fields were selected in each slide to calculate the number of positive cells.

### 2.10. Quantitative Real-Time PCR Analysis of miR-21

Blood samples for miRNA detection were collected and centrifuged to spin down the blood cells. Total RNA was isolated from the plasma using mirVana RNA isolation kit according to the manufacture instructions. RNA was transcribed reversely to cDNA using TaqMan® MicroRNA Reverse Transcription kit. Rno-miR-21 sequence was UAGCUUAUCAGACUGAUGUUGA and internal reference rno-miR-191a-5p sequence was CAACGGAAUCCCAAAAGCAGCUG. The miR-21 expression levels were measured quantitatively using the stem-loop/TaqMan probe method. The cycle threshold (Ct) was automatically given by ABI 7500 System Sequence Detection software 1.2. After normalization, CT values were converted to relative gene expression levels using the 2^−ΔΔCT^ method.

### 2.11. Statistical Analysis

Data were expressed as mean ± SD. All statistical analyses were performed with SPSS statistics software (version 20.0, SPSS Inc., Chicago, IL, USA). The differences of various measurements among groups were determined by one-way ANOVA and the difference with* P* value <0.05 (two-tailed) was considered significant.

## 3. Results

### 3.1. SKSF Decreased FBG and HbA1c Levels

**Figure 1:**
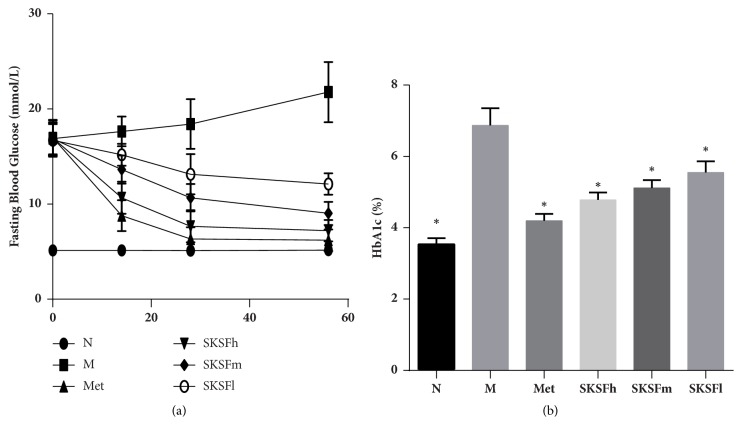
SKSF decreased FBG and HbA1c levels. (a) Fasting blood glucose, (b) HbA1c. Data were expressed as mean ± SD, n* *=10 rats per group. N, normal group; M, model group; Met, metformin group; SKSF-l, Spleen-Kidney Supplementing Formula group at a low dose; SKSF-m, Spleen-Kidney Supplementing Formula group at a medium dose; SKSF-h, Spleen-Kidney Supplementing Formula group at a high dose. ^*∗*^*P* <0.05 compared with model group.

FBG levels in group M remained significantly high throughout the experiment. On days 14, 28, and 56 of drug administration, FBG levels descended significantly and gradually in all treatment groups (*P *< 0.05). In particular, the hypoglycemic effect was more obvious in Group Met and Group SKSF-h (Figure 1(a)). The HbA1c levels, another indicator of blood glucose concentration, were also significantly lower in the SKSF treatment groups compared with those in group M (*P* < 0.05) (Figure 1(b)) (the impact of SKSF on body weight and food intake was presented in Supplement 1).

### 3.2. SKSF Improved BUN, UA, Urea, CCR, KW/BW, and 24-h Urine Protein

**Figure 2:**
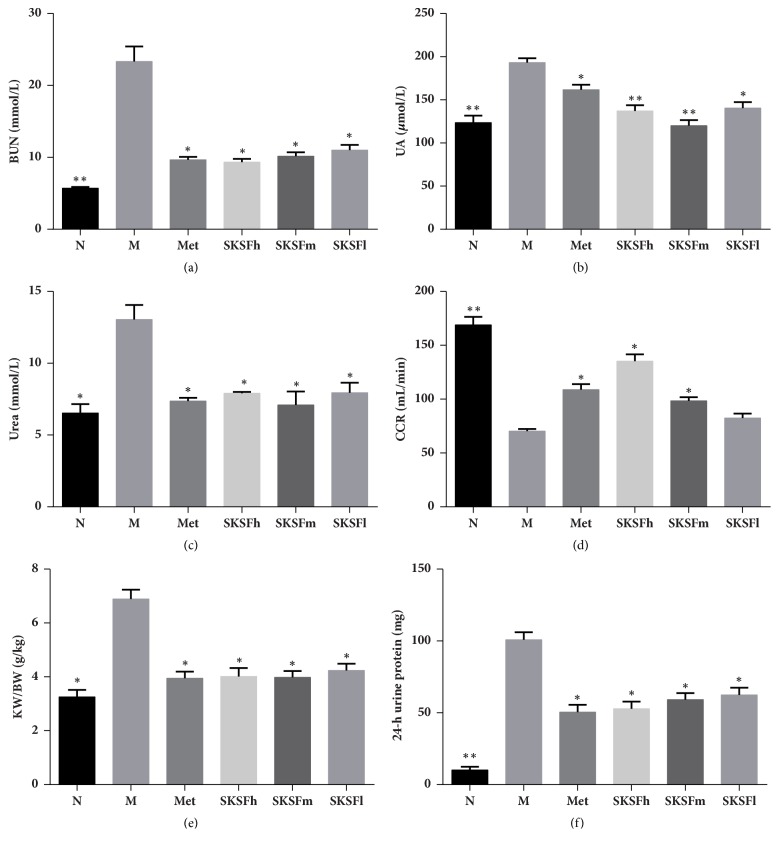
SKSF improved BUN, UREA, UA, CCR, KW/BW, and 24-hour urine protein in T2DM rats. (a) BUN, (b) UA, (c) Urea, (d) CCR, (e) KW/BW, (f) 24-hour urine protein. Data were expressed as mean ± SD, n* *=10 rats per group. N, normal group; M, model group; Met, metformin group; SKSF-l, Spleen-Kidney Supplementing Formula group at a low dose; SKSF-m, Spleen-Kidney Supplementing Formula group at a medium dose; SKSF-h, Spleen-Kidney Supplementing Formula group at a high dose. ^∗^*P* < 0.05, ^∗∗^*P* < 0.01 compared with model group.

In T2DM rats, the concentrations of BUN, UA, urea, and 24-hour urine protein excretion were significantly higher (*P *< 0.05), and the KW/BW ratio was also significantly elevated (*P *< 0.05), while creatinine clearance rate (CCR) decreased. SKSF significantly decreased the concentrations of BUN, UA, urea, CCR, 24-hour urine protein excretion, and the KW/BW ratio (*P *< 0.05) and increased CCR (Figure 2).

### 3.3. SKSF Improved the Morphology and Structure of Renal Tissue

**Figure 3:**
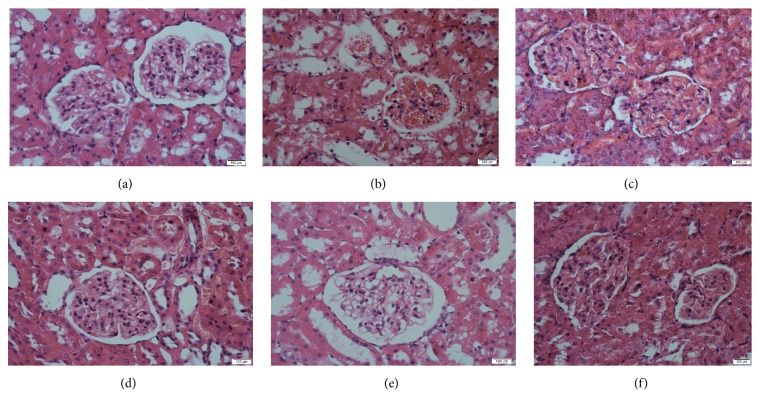
SKSF improved the morphology and structure of renal tissue. (a) Normal group, (b) model group, (c) metformin group, (d) Spleen-Kidney Supplementing Formula group at a high dose, (e) Spleen-Kidney Supplementing Formula group at a medium dose, (f) Spleen-Kidney Supplementing Formula group at a low dose.

Compared to the group N, renal morphological changes including basal membrane thickening and vacuolar degeneration in the renal tubular epithelial cells, inflammatory cells infiltration within renal interstitium, and renal interstitium enlargement were observed in the group M (Figure 3(b)). However, in the SKSF groups, these changes were reversed to some degree (Figures 3(d)–3(f)).

### 3.4. SKSF Attenuated the Renal Fibrosis

**Figure 4:**
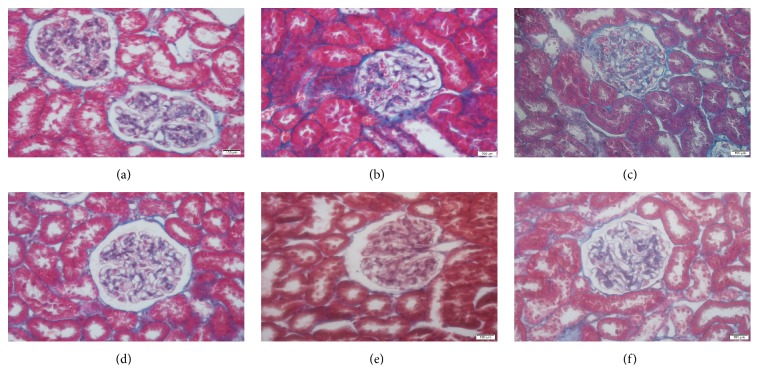
SKSF attenuated the renal fibrosis. (a) Normal group, (b) model group, (c) metformin group, (d) Spleen-Kidney Supplementing Formula group at a high dose, (e) Spleen-Kidney Supplementing Formula group at a medium dose, (f) Spleen-Kidney Supplementing Formula group at a low dose.

As shown in Figure 4, Masson's trichrome staining revealed collagen deposition (blue color) in the interstitium. Collagen was stained in blue. In group M, glomeruli and the tubular epithelium were strongly collagen staining positive, suggesting obvious glomerular and interstitial fibrosis in T2DM rats. After intervention, the scope of collagen-positive stained area was narrowed and the degree of positive staining was weakened, indicating collagen deposition was alleviated in renal tissues (Figure 4).

### 3.5. Influence of SKSF on the Expressions of TGF-*β*1 and PTEN

**Figure 5:**
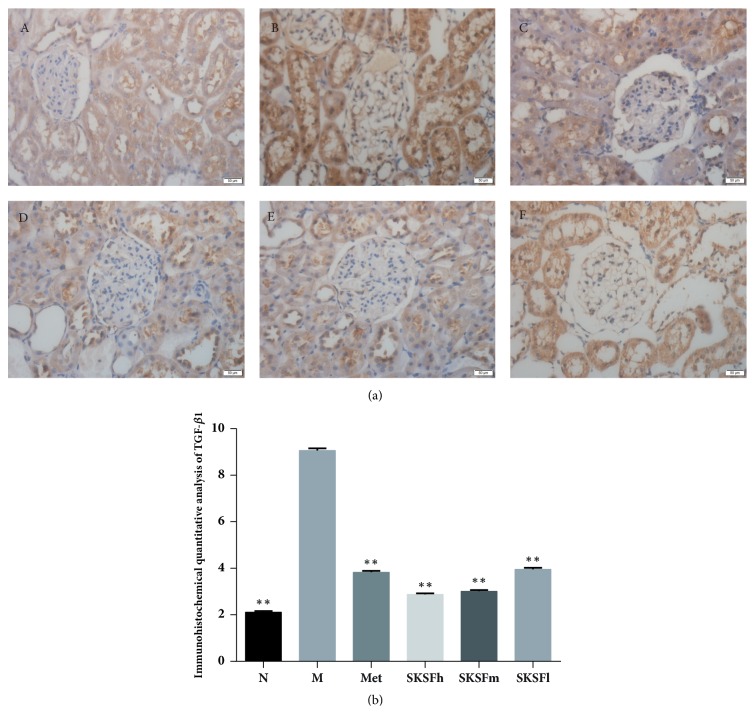
Influence of SKSF on TGF-*β*1 expression. (A) Normal group, (B) model group, (C) metformin group, (D) Spleen-Kidney Supplementing Formula group at a high dose, (E) Spleen-Kidney Supplementing Formula group at a medium dose, (F) Spleen-Kidney Supplementing Formula group at a low dose. Data were expressed as mean ± SD, n* *=10 rats per group. ^∗^*P* < 0.05, ^∗∗^*P* <0.01 compared with model group.

In group N, renal tissue was stained light brown and counterstained with hematoxylin (blue). In group M, the glomerular mesangial cells and epithelial cells of Bowman's capsule were stained strongly positive, suggesting TGF-*β*1 expression was obviously high (*P* < 0.01). Massive and flake-shaped staining was localized to the cytoplasm. After intervention, the degree of positive staining was weakened, indicating significant decrease of TGF-*β*1 expression (Figure 5).

**Figure 6:**
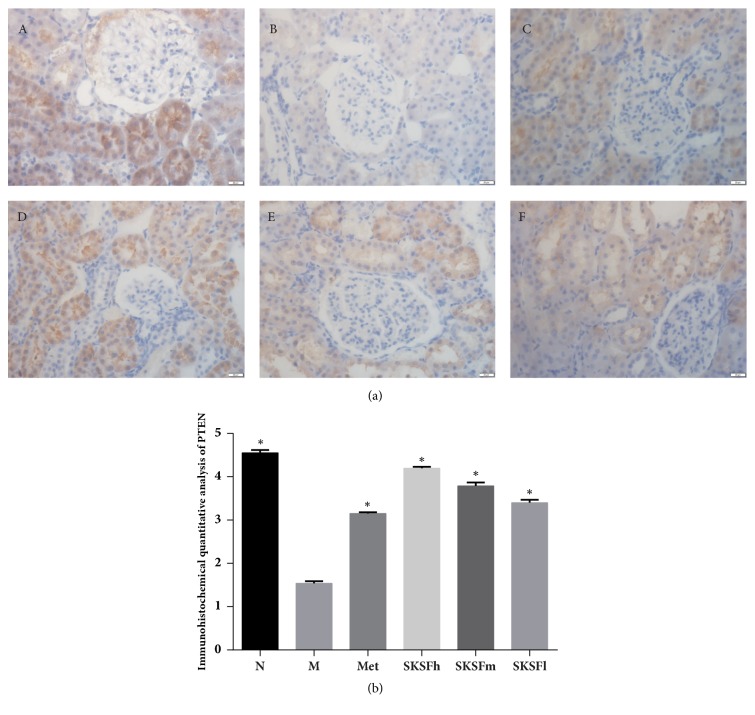
Influence of SKSF on PTEN expression. (A) Normal group, (B) model group, (C) metformin group, (D) Spleen-Kidney Supplementing Formula group at a high dose, (E) Spleen-Kidney Supplementing Formula group at a medium dose, (F) Spleen-Kidney Supplementing Formula group at a low dose. Data were expressed as mean ± SD, n* *=10 rats per group. ^∗^*P* < 0.05 compared with model group.

PTEN is mainly located in renal tubular epithelial cells (brown color). In group N, the brown staining was strongly positive. But the brown staining was weakened obviously in group M, suggesting the PTEN expression was in a significant reduction (*P* < 0.05). After treatment, positive staining was strengthened to some degree, indicating PTEN expression was increased remarkably (Figure 6).

### 3.6. Effects of SKSF on the mRNA Expression of Mir-21

**Figure 7:**
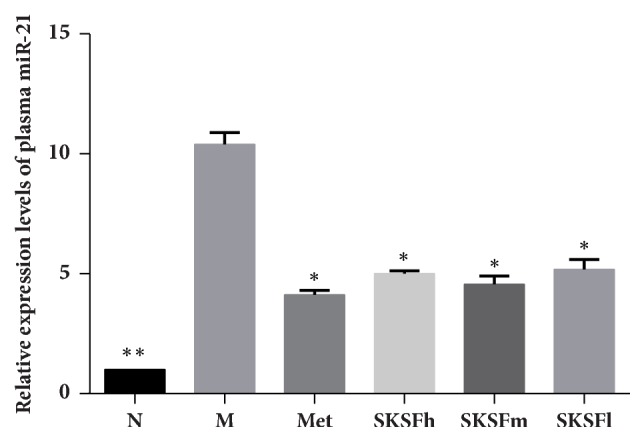
Effects of SKSF on the mRNA expression of miR-21. Data are expressed as mean ± SD, n* *=10 rats per group. (N) Normal group, (M) model group, (Met) metformin group, (SKSFh) Spleen-Kidney Supplementing Formula group at a high dose, (SKSFm) Spleen-Kidney Supplementing Formula group at a medium dose, (SKSFl) Spleen-Kidney Supplementing Formula group at a low dose. ^∗^*P* < 0.05, ^∗∗^*P* <0.01 compared with model group.

Compared with group N, the relative expression of plasma miR-21 in T2DM rats was significantly increased, while SKSF could decreased the miR-21 expression (Figure 7).

## 4. Discussion

In this study, high-fat diet combined with multiple low-dose STZ injections was used to establish T2DM rat model. At the end of the experiment, the concentrations of FBG, HbA1c, BUN, UA, urea, 24-hour urine protein excretion, and KW/BW ratio increased significantly, while CCR decreased in T2DM rats. H&E staining shows thickening of the basal membrane and vacuolar degeneration in the renal tubular epithelial cells, infiltration of inflammatory cells within renal interstitium, and the enlargement of renal interstitium in T2DM rats. Masson's trichrome staining revealed obvious glomerular and interstitial fibrosis in model rats. The changes of physiological indicators indicate an obvious renal fibrosis and an early sign of DN in T2DM rats. After the treatment with SKSF for 8 weeks, the symptoms of diabetic rats were relieved, the levels of FBG and HbA1c were reduced, the renal function was significantly improved, and the pathologic changes like renal fibrosis and collagen deposition in renal interstitium were alleviated. These observations confirmed the effectiveness of SKSF in the treatment of T2DM and DN.

Transforming growth factor-beta (TGF-*β*) is a pleiotropic cytokine with potent regulatory and inflammatory activity. TGF-*β* is not only involved in the growth and differentiation of various cells, such as liver cells, kidney cells, and heart cells, but also involved in the synthesis and accumulation of extracellular matrix (ECM), which can lead to renal interstitial fibrosis and even glomerular sclerosis. TGF-*β* promotes transformation of fibroblast and ECM synthesis and inhibits ECM decomposition by regulation on matrix metalloproteinase, which not only leads to podocytes apoptosis, basement membrane denudation, and protein leakage, but also reduces nephrons (functional unit of kidney) and even glomerular sclerosis [16]. In this study, the results of immunohistochemistry on renal tissues show that the expression of TGF-*β* was significantly elevated. SKSF can obviously reduce TGF-*β* expression, which may act as one of the mechanisms to alleviate the renal fibrosis.

miR-21 has been proved to be one of the important microRNAs involved in in renal fibrosis. Many studies have shown that elevated TGF-*β* expression in renal tubular epithelial cells was observed in a high-sugar environment. Through TGF-*β*/Smad3 signaling pathway, TGF-*β* promotes miR-21 expression, which induces cell damage and leads to phenotypic transformation [17, 18]. In present study, plasma miR-21 was significantly increased in the diabetic rats at an early stage of DN. SKSF can significantly reduce the expression of miR-21.

Relevant studies have confirmed that PTEN, one of the targets of miR-21, is decreased under a high glucose condition. Decreased PTEN function leads to overactivation of PI3K/Akt [19, 20]. The abnormal activation of PI3K/AKT signaling pathway triggers a downstream cascade of a variety of enzymes and transcription factors through phosphorylation. Inhibition of PI3K/AKT signaling pathway indirectly leads to reduction of *α*-SMA and collagen IV, protects renal tissues, and delays the progression of DN [21]. In this study, results by immunohistochemistry show that SKSF reverses the structural and functional abnormality of renal tubules in early T2DM rats. The mechanism may be associated with expression of PTEN increase in renal tubules in diabetic rats.

## 5. Conclusion

Preliminary experiments reveal that SKSF exerts a positive effect on glycolipid metabolism [22]. In present study, SKSF significantly reduced the concentrations of BUN, UA, urea, 24-hour urine protein excretion, and the KW/BW ratio, increased CCR, and attenuated the pathological changes and renal fibrosis in T2DM rats. SKSF can relieve early DN, which may be associated with its regulation via TGF-*β*1-miR-21-PTEN signaling pathway.

## Data Availability

The data used to support the findings of this study are available from the corresponding author upon request.

## Supplementary Materials

Supplementary Materials Supplement 1. The effect of SKSF on body weight and food intake: (a) body weight, (b) food intake. Data were expressed as mean ± SD, n* *=10 rats per group. N, normal group; M, model group; Met, metformin group; SKSF-l, Spleen-Kidney Supplementing Formula group at a low dose; SKSF-m, Spleen-Kidney Supplementing Formula group at a medium dose; SKSF-h, Spleen-Kidney Supplementing Formula group at a high dose. ^∗^*P*<0.05 compared with model group.
