# Supplementary material for: Spleen-Kidney Supplementing Formula Alleviates Renal Fibrosis in Diabetic Rats via TGF-β1-miR-21-PTEN Signaling Pathway
Source: Evid Based Complement Alternat Med. 2018 Dec 6;2018:3824357. doi: 10.1155/2018/3824357 (PMC6304600; doi:10.1155/2018/3824357)
Supplement: Supplementary Materials — Supplement 1. The effect of SKSF on body weight and food intake: (a) body weight, (b) food intake. Data were expressed as mean ± SD, n =10 rats per group. N, normal group; M, model group; Met, metformin group; SKSF-l, Spleen-Kidney Supplementing Formula group at a low dose; SKSF-m, Spleen-Kidney Supplementing Formula group at a medium dose; SKSF-h, Spleen-Kidney Supplementing Formula group at a high dose. ∗P<0.05 compared with model group. [file 3824357.f1.pdf]

Spleen-kidney Supplementing Formula Alleviates Renal Fibrosis in Diabetic Rats via TGF-β1- miR-21-PTEN signaling pathway

Chunyu Tian[1*#], Ya Wang[1 #], Hong Chang [1#], Ji'an Li[1], Xiaojin La[1]

1. Traditional Chinese Medical College, North China University of Science and Technology, Tangshan 063210, China

Correspondence should be addressed to Chunyu Tian, tcy4479@sina.com.

#: These authors contributed to the paper equally.

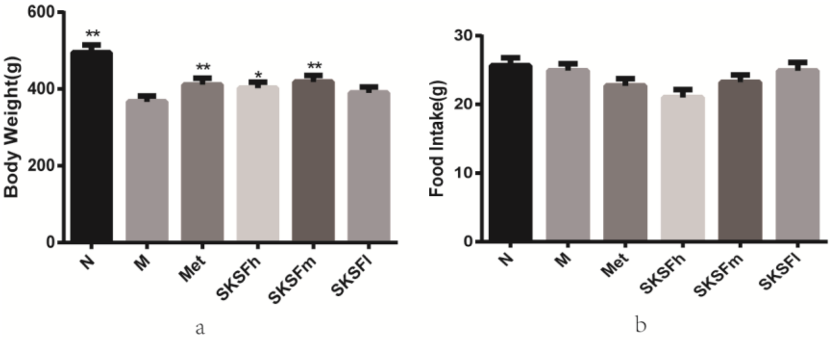

Supplement 1: The effect of SKSF on body weight and food intake. a Body weight, b Food intake. Data were expressed as mean ± SD, n=10 rats per group. N, normal group; M, model group; Met, metformin group; SKSF-l, Spleen-kidney Supplementing Formula group at a low dose; SKSF-m, Spleen-kidney Supplementing Formula group at a medium dose; SKSF-h, Spleen-kidney Supplementing Formula group at a high dose. * P <0.05 compared with model group.
